# Mast Cells in Human Health and Diseases 2.0

**DOI:** 10.3390/ijms25126443

**Published:** 2024-06-11

**Authors:** Giovanna Traina

**Affiliations:** Department of Pharmaceutical Sciences, University of Perugia, Via Romana, 06126 Perugia, Italy; giovanna.traina@unipg.it

## 1. Introduction

This Special Issue collects some scientific pieces of the multifaceted research on the mast cell (MC), and it intends to highlight the broad spectrum of activity that MCs have, both in physiological conditions and in pathological states, focusing attention on some of them.

The present Special Issue is a continuation of a previous Special issue on MCs, entitled “Mast cells in Human Health and Diseases”, published by the *International Journal of Molecular Sciences* [1].

## 2. Mast Cells

MCs are cells of hematopoietic origin of innate and adaptive immunity, heterogeneous and multifunctional cells, present extensively in mucosal and epithelial tissues throughout the body [2,3]. In recent decades, a growing number of studies have been dedicated to dissecting the roles and peculiarities of MC actions, and evidence has confirmed that MCs play a crucial role in human health and diseases [4,5,6].

In the physiological context, MCs regulate many processes, including vasodilation, vascular homeostasis, innate and adaptive immune responses, homeostasis of the immune system, the immune landscape of cancer, angiogenesis, and lymphangiogenesis [2].

On the pathological side, MCs are intriguing and multifactorial cells, notoriously known to be involved in allergic responses, asthma, anaphylaxis, and other disorders, including gastrointestinal disorders, through degranulation and the release of a variety of mediators with vasoactive, inflammatory, and nociceptive activities [7,8].

MCs can be isolated and differentiated from a wide variety of tissues when integrated with stem growth factor and interleukins 3 and 6. In particular, it has emerged that important differences in the phenotypic markers of MCs derive precisely from the conditions of differentiation, donor, and cultivation medium [9].

Mast cells are sensors of the environment, able to respond promptly and selectively to a wide variety of stimuli. The responses of MCs to different stimuli are extremely heterogeneous and can be conditioned by the microenvironment and intrinsic factors. They can also modify the expression or functionality of both receptors and signaling molecules [10].

MCs have a broad repertoire of receptors. Pattern recognition receptors (PRRs) are involved in the nonspecific detection of foreign pathogens. Toll-like receptors (TLRs) are a subclass of PPRs and play a role in innate immunity and inflammatory responses, and MCs express most TLR receptors [11,12,13].

These characteristics allow MCs to act as sentinels for the host defense against pathogens and respond to metabolic and immune changes in the microenvironment in which they reside.

Furthermore, mast cells communicate with many immune and non-immune cells [14].

After activation, they release a variety of molecules, which include histamine, leukotrienes, and prostanoids, as well as proteases, chemokines, and cytokines, fundamental in the genesis of the inflammatory response. MCs can also release extracellular vesicles and extracellular DNA traps.

MCs have some functions common to those of professional phagocytes. Given their location and the mediators produced, MCs play an active role in many pathologies, including infections, arthritis, tumors and pulmonary fibrosis. Since MCs are a source of fibrogenic cytokines (TGF-β and PDGF and β-FGF), they are also associated with various fibrotic pathologies [13].

The localization of MCs within the tissue and their expression of specific mediators are important pieces of evidence from a diagnostic point of view and as an aggravating factor of a pathological state. For example, histamine degranulation of cochlear MCs could contribute to exacerbating the toxicity of the chemotherapeutic agent cisplatin [15].

In this context, tryptase contributes to osteoarthritic (OA) pathology [16], and tryptase genes were highly expressed in the synovium of overweight OA patients [17]. In addition, an interesting correlation has been seen between the localization of MCs and osteoclasts. Studies revealed that MC-deficient mice are protected from bone loss and osteoclastogenesis [18]. MCs were shown to produce the receptor activator of the NF-κB ligand (RANKL), the key factor regulating osteoclast formation. It has also been reported that MC-derived RANKL is not involved in the pathology of post-menopausal osteoporosis [19,20].

MCs release a plethora of factors involved variously in inflammation, pain, fibroblast proliferation, and angiogenesis—processes known to be involved, among other things, in the pathogenesis of endometriosis [21,22]. Evidence reports that MCs, under the influence of estrogen, are recruited to the endometriotic lesion microenvironment and play an active role in endometriosis pathophysiology [23,24].

Chronic inflammation is a common mediator of various disorders, which include many pathological conditions, pain, migraine, depression, anxiety, autoimmune diseases, and fibromyalgia, as well as Alzheimer’s disease, multiple sclerosis, atherosclerosis, cardiovascular diseases, and skin diseases, such as eczema and atopic dermatitis [25,26].

Finally, MCs are also implicated in COVID-19, as the virus activates these cells, which release various pro-inflammatory molecules [27,28].

MCs are an important component of the gastrointestinal tract, influencing immune and pathological processes affecting the digestive tract [3,7]. It is known that the physiological functioning of the intestinal epithelial barrier is crucial for maintaining the homeostasis of the internal medium, while uncontrolled barrier mechanisms could lead to an increase in mucosal permeability and the passage of luminal antigens, toxins, and/or microorganisms through the intestinal epithelium, potentially induce disturbances in epithelial–neuro–immune interactions, and facilitate and exacerbate inflammatory states [29,30].

A compromised epithelial barrier is implicated in the origin and development of many digestive and non-digestive diseases. Therefore, the control of intestinal permeability represents a central mechanism in the treatment and prevention of human diseases. An altered function of MCs can interrupt the function of the intestinal epithelial barrier by changing the permeability of the mucosa. Mast cell products, on the other hand, may contribute to hypersensitivity phenomena [30].

Evidence reports the role of specific bacterial strains in contributing to the maintenance of the integrity of the intestinal epithelial barrier [7,31]. Studies have also been conducted on the ability of bacterial strains to intervene in the dialogue between the intestinal barrier and MCs [32,33].

It has been suggested that the stabilization of MCs as well as the control of cytokine release, for example using natural substances, such as resveratrol or probiotics, may constitute valid and promising therapeutic tools [3,26,30,33].

The modulation of the hyperactivity of MCs and the reduction in the release of inflammatory factors could constitute new frontiers of therapeutic interventions aimed at preventing chronic inflammation.
